# Characteristics of Korean Children and Adolescents Who Die by Suicide Based on Teachers’ Reports

**DOI:** 10.3390/ijerph19116812

**Published:** 2022-06-02

**Authors:** Mi-Sun Lee, Jin Ho Jhone, Joon Beom Kim, Yong-Sil Kweon, Hyun Ju Hong

**Affiliations:** 1 Department of Preventive Medicine, College of Medicine, The Catholic University of Korea, Seoul 06591, Korea; dlaltjs1010@hanmail.net; 2 Department of Social Welfare, Soongsil University, Seoul 06978, Korea; headcr@yonsei.ac.kr; 3 Interdisciplinary Graduate Program in Social Welfare Policy, Yonsei University, Seoul 03722, Korea; bokjiman@kakao.com; 4 Department of Psychiatry, Uijeongbu St. Mary’s Hospital, College of Medicine, The Catholic University of Korea, Seoul 06591, Korea; yskwn@catholic.ac.kr; 5 Department of Psychiatry, Hallym University Sacred Heart Hospital, Anyang 14068, Korea; 6 Suicide and School Mental Health Institute, Hallym University, Anyang 14068, Korea

**Keywords:** suicide, death, students, characteristics, adolescent, school, teachers, Korea

## Abstract

We analyzed the clinical characteristics and suicide-related factors of students who died by suicide in Korea in 2016–2020, based on teachers’ reports. Using data on total suicide deaths (N = 654, mean age = 16.0, 52.6% boys) collected by the Ministry of Education of Korea, we investigated the demographic and clinical characteristics and suicide-related factors of suicide deaths in students aged 9–18 years. Considering gender, more boys (52.6%, N = 344) died by suicide than girls (47.4%, N = 310). About 425 (65.0%) of the suicides were among high school students. The most common suicide method was jumping from a high place (70.6%, N = 454), followed by hanging (25.7%, N = 165). Additionally, 9.4% (N = 48) of the students who died by suicide had a history of attempted suicide, 13.4% (N = 73) had attempted self-harm, and 12.8% (N = 48) were acquainted with someone who had died by suicide. Teachers observed 20.6% (N = 120) of the suicide warning signs at school. Girls tended to have higher rates of attempted suicide, emotional problems, and psychiatric disorders than boys. This study was conducted with the largest sample of Korean suicide students observed at school. Effective suicide-related mental health training for teachers could contribute to suicide prevention in students.

## 1. Introduction

According to the World Health Organization (WHO), suicide was the fourth leading cause of mortality among 15–19-year-olds adolescents globally in 2019 [1,2,3]. Additionally, suicide accounts for 8.5% of all deaths among children, adolescents, and young adults (15–29 years old) [4,5]. Between 2000 and 2019, across the Organization for Economic Cooperation and Development (OECD) countries, deaths by suicide fell overall by 29% [6,7,8]. Moreover, rate of death by suicide per 100,000 population decreased or remained in most OECD countries [7]. In contrast, in Korea, deaths by suicide increased by 46%, and suicide rates were higher (24.6 per 100,000) than in other countries from 2000 to 2019 [9]. Additionally, in the United States, suicide rate was a stable period from 2000 to 2007. However, from 2007 to 2018, suicide rate among adolescents and young adults (aged 10–24) increased by 57.4% (6.8 per 100,000) [10]. Furthermore, according to a report on the causes of death statistics in Korea published in 2020, Korean adolescents (aged 10–19) who died by suicide in 2020 also accounted for 6.5 per 100,000 of the population [11].

Based on global all-cause deaths and disability-adjusted life years (DALYs) for 2019 with trends [12], the average suicide rate for children and young adults aged 0–19 in OECD countries was 1.70. The average for Korea was 2.13, which ranks Korea as the top 10 among 38 countries. In OECD countries, the suicide rate increases with age and is higher among men. The average suicide rate for those aged 0–19 was 2.43 for men and 0.93 for women in 2019. In Korea, as a result of the subdivision by gender, the suicide rate of boys was 2.40, which was lower than the average, but 1.84 for girls, which was about twice the average [12].

The causes and risk factors for suicide in children and adolescents is heterogeneous and influenced by individual, family, and school-related aspects [13,14]. Moreover, it is associated with several risk factors, such as early childhood adversities (e.g., emotional neglect, bullying, and sexual abuse), academic shortcomings, and mental illness (e.g., low self-esteem, impulsivity, aggression, hopelessness, sleep disturbances, substance use, antisocial behavior, post-traumatic stress disorder, and affective mood disorders) [5,15,16,17,18,19]. Psychopathological issues have shown negative effects on school performance, peer relationships, well-being, and mental health problems in adolescence, and it might affect the onset of other psychiatric illnesses [3,11].

Sociodemographic factors might increase the risk for dying by suicide [20]. Socioeconomic status is linked to poor mental health in adolescents in terms of suicide [18]. Previous research has shown that adolescents from highly educated families have lower risk of depressive symptoms, anxiety, and suicide attempts [21]. Increased vulnerability to domestic environmental factors (parental psychopathology, familial conflict, parental education levels, parental divorce, and a family history of suicide) and economic status might affect pathways that lead adolescents to suicide [22,23]. Various factors and conditions can become trigger events for suicidal behavior because stressful life events (e.g., interpersonal problems) often precede suicidal behavior in children and adolescents [2,20]. Nevertheless, direct associations with risk factors of child and adolescent suicide are difficult to identify [24,25].

Suicide death in children and adolescents is a rare event, so many studies on child and adolescent suicide have focused on suicidal ideation or suicide attempts. However only 22% of suicide deaths have a history of suicide attempts, [26] and considerable differences might exist between suicide attempts and suicide deaths.

Particularly, students spend most of their time at school, and schools are places that create a safe environment for students [10]. Additionally, conducting suicide prevention education is useful as it is easier to detect students’ mental health problems in schools [2]. Therefore, school-based suicide prevention policy is considered one of the most effective ways to address the problem of children and adolescent’s suicide. Above all, teachers are crucial in students’ development and can also act as gatekeepers for students’ suicide prevention [27].

Therefore, this study aims to investigate the clinical characteristics, demographics, and suicide-related factors of students who have died by suicide based on teachers’ reports collected by the Ministry of Education of Korea during 2016–2020. We also compare differences in suicide characteristics between boys and girls.

## 2. Methods

### 2.1. Database

Our analysis is based on a national database comprising data collected through schools on students who had died by suicide after receiving the permission of the Ministry of Education of Korea. When students die by suicide, their teachers submit a student suicide report to the Ministry of Education. Student suicide reports, which include teachers’ observations, parental reports regarding the circumstances of the death, and information collected by the school as an official education record, have been collated as part of Korea’s national student suicide prevention policy since 2015.

This questionnaire form was developed by the Suicide and School Mental Health Institute, Hallym University, Korea. During development, final survey items were composed by referring to previous studies and opinions of officials from the Ministry of Education and are being continuously revised. The previous study described the detailed survey items [3].

Data on students from the second grade of elementary school to the third grade of high school, corresponding to the ages 9–18 years, were included. In this study, we used national data collected from 2016 to 2020 and analyzed 654 student suicides during that period.

### 2.2. Variables

We analyzed students’ sociodemographic variables, including gender, school type, family structure, socioeconomic status, factors related to suicide (place of suicide, method of suicide, history of attempted suicide and self-harm, experience of an acquaintance’s death by suicide, and warning signs noticed by teachers). The questionnaire with warning signs for students who died by suicide was based on contents presented by the Korea Psychological Autopsy Center [28]. Mental health problems (psychological problems, physical illness, and mental illness) based on teachers’ responses to a “yes/no” questionnaires were also included. School-related variables (attendance and school acceptance) were also analyzed. School acceptance represented a sense of belonging to school as perceived by teachers. This was rated on a scale of 1 to 4, with higher scores being interpreted as positive. The questionnaire included concerns raised at school in the year prior to the suicide death of a student. Unusual concerns revealed at school within 1 year prior to death by suicide were classified into five items, which included individual, family, peer, learning, and addiction problems. Multiple responses were allowed for associated concerns raised at school.

### 2.3. Statistical Analysis

Data analysis was conducted using SPSS 23.0 (IBM) (Chicago, IL, USA) for descriptive statistical analysis. The Chi square test, Fisher’s exact test, and t-test were used for gender-based comparisons by variables (sociodemographic variables, factors related to suicide, mental health problems, School-related variables, and unusual concerns revealed at school within 1 year prior to death by suicide). We used a significance level of 0.05 for the statistical test.

## 3. Results

### 3.1. Descriptive Statistics

In Korea, 654 students (Mean age = 16.0, SD = 1.7) died by suicide from 2016 to 2020. On gender, more boys (52.6%) than girls died by suicide. The most common place of suicide was home (62.1%). Additionally, the most common suicide method was by jumping from a high place (70.6%). Prior to death by suicide, 40% of students had reported psychological problems, 18.7% had complained of a physical illness, and 24.4% had complained of a mental illness. Additionally, 47.9% had experienced a trigger event (e.g., death of a close person, conflict with others) immediately before their death by suicide. Only 9.4% had a past history of attempted suicide, 13.4% had attempted self-harm. Table 1 summarized the demographic characteristics.

### 3.2. Usual Concerns Revealed at School within One Year Prior to Death by Suicide

We examined the concerns of students who died by suicide. These concerns were expressed in the year before their death. Teachers heard about the concerns from the subjects or their friends or observed them at school. In particular, 44% of the students showed no apparent concerns. Family problems were the most frequently observed (53.8%), followed by academic problems, individual problems, peer problems, and addiction problems. Table 2 summarizes the findings.

### 3.3. Comparison of Gender Differences of Students Who Died by Suicide

After examining gender differences among Korean students who died by suicide, various factors (method of suicide, psychological problems perceived by teachers, mental illness, trigger event before suicide, history of attempted suicide or self-harm, warning signs of suicide, school attendance, and individual/family/learning/addiction problems) showed statistically significant differences (Table 3).

### 3.4. Comparison of Gender Differences Related to Usual Concerns

The analysis revealed gender differences related to usual concerns revealed at school within one year prior to the students’ death by suicide. In terms of factors for mental health problems in individual problems, girls were more likely to have mental health issues than boys, and there was a statistically significant difference (X^2^ = 18.692, *p* < 0.001). Boys were more likely to have game addiction problems than girls, and there was a statistically significant difference (X^2^ = 22.607, *p* < 0.001). There were also statistically significant differences in conflict between parent and child in family problems (X^2^ = 5.588, *p* < 0.05), and the burden of a competitive school environment in academic problems (X^2^ = 6.717, *p* < 0.05). The results are summarized in Table 4.

## 4. Discussion

This study investigated the clinical characteristics, demographic, and suicide-related factors of Korean students who died by suicide based on teachers’ reports and compared the differences between boys and girls. The study’s sample size was very large compared to previous studies [29,30] on children and adolescents who died by suicide. It was the largest sample of Korean students observed at school and can be considered representative of Korean students who have died by suicide.

This study’s finding that suicide rates increase with age is consistent with previous studies [3,11], and could be attributable to the increasing number of risk factors for suicide, such as depression. Globally, suicide in adolescent boys is higher than in girls, with a gender ratio of 2–3:1 [31]. In our study, although boys had a higher suicide rate than girls, it was not statistically significant. This means that the suicide rate among female Korean students is higher than in other countries. Particularly, Korea has many high-rise buildings, so suicide access can be easier compared to other countries [32]. Therefore, even girls, who are less impulsive than boys, are just as prone to suicidal behavior [3].

This study also found a statistically significant difference in the characteristics of suicide according to gender. Studies in other countries have indicated that girls report more emotional symptoms (e.g., depression) and interpersonal conflict, while boys are more often associated with engagement in violent experiences and anger-related symptoms [2,33,34] The current study’s findings are similar to those of earlier studies [35] and confirm that the effects of risk factors differ by gender. Boys tend to show more externalized problems (anger, difficulties with impulse control, risk-taking, and physical aggression) than girls, whereas girls tend to show more internalized problems (depressive symptoms and social exclusion) than boys [36]. However, gender differences in suicide could also be associated with psychosocial differences or cultural acceptability [37].

Our study found that jumping from a high place was the most common suicide method. South Korea, a densely populated country, has many residents in high-rise buildings. Therefore, most suicides by jumping from high places in South Korea have occurred in high-rise residential housing units [38]. Furthermore, prior studies indicated that suicide by jumping from a high place was the most common method of suicide, accounting for 56 percent of suicides among Korean children and adolescents aged 10 to 19 [39]. The reason for this is unclear, but easy access could be a contributing factor. Considering previous findings, children and adolescents who attempt acts of high medical lethality, such as hanging or jumping, are at extremely high risk of dying by suicide [40]. Lethal means have high case-fatality rates and provide limited opportunity for crisis intervention. The creation of safe surroundings among those at risk of suicide could be a successful strategy for preventing suicide [41].

However, teachers only noticed 20.6% of students’ warning signs before death by suicide at school. Teachers also reported that 42.1% of the problems were psychological, 24.4% were related to psychiatric disorders, 13.4% were self-harm, and 9.4% were suicide attempts. Previous studies have also shown that more than 90% of the adolescents who died by suicide were diagnosed with a psychiatric disorder and that more than 90% showed signs of suicide warning [24]. This study found that psychiatric problems are not well revealed before the death of children and adolescents who died by suicide. Psychiatric problems may go unnoticed in schools as the deceased students had not previously expressed their inner feelings or thoughts. Additionally, our results demonstrated that higher emotional problems were reported in girls than in boys by teachers. This could be linked to overall greater acceptability of emotional expression among girls than boys. Particularly, we found that teachers general had difficulty in noticing personal and emotional problems of boys. Therefore, we should consider and observe the expression of inner feelings by gender or characteristics in the school setting.

In this study, 42% of children and adolescents who died by suicide had psychological problems, and 56% had apparent concerns during the year before their death by suicide but without impacting their school attendance or acceptance level at school. Among the students’ usual concerns observed by teachers in the year before their death, family problems were the highest at 53.8%, followed by academic problems (46.4%), and individual problems (36.5%), including psychiatric disorders.

This study did not utilize a control group, and data were based on teachers’ reports after the death of the student and did not obtain specific information on the content and extent of usual concerns. Therefore, whether or not the usual concerns of the deceased were related to risk factors or triggers of suicide is unclear. However, as suicide is not an acute accident caused by an external factor, but a complex and continuous process wherein various risks and protective factors are interrelated, usual concerns may be related to risk factors for suicide. This study suggests a need to respond more sensitively to students at risk and perform a suicide assessment when encountering students who have psychological difficulties at school, even if the intensity does not appear to be severe.

Positive interactions and personal relationships might be useful in maintaining emotional mental health and buffering the stressors [31]. Absence of reliable relationships may increase children and students’ vulnerability to negative stressful events and consequently their risk of suicidal behaviors [42]. Students develop socially and emotionally through their interactions with teachers and peers at school. In this study, school acceptance of suicide students was good. Teachers may be in a position of reliable person for students at risk of suicide. Also, effective implementation of suicide warning signals and suicide-related mental health education for teachers will contribute to the prevention of suicide in children and adolescents. Furthermore, our results could suggest training and strengthening of the presence of structured personnel in schools with psychological supportive roles (e.g., psychologists, psychotherapists, and counselors). Furthermore, teachers should receive training to develop their emotional and social abilities.

In particular, South Korea has undergone rapid social change in recent decades and achieved economic growth, ranking 11th in the world in terms of gross domestic product (GDP) in 2018 [43]. Owing to the competitive social situation in Korea where achievement and academic performance are important, intergenerational conflict is emerging as a major social problem. Moreover, suicide rate among children and adolescents has been continuously increasing in recent years [44].

This study has a few limitations. The present results were limited to data included in teachers’ reports. Moreover, this data were collected only retrospectively when teachers’ reports could be biased by suicidal events. Therefore, these reports might not fully reflect all the information known about students’ suicide. This study did not include a control group. We presented only the descriptive analysis of the basic sociodemographic characteristics and risk factors of students who died by suicide. Thus, further in-depth studies should be needed to focus on the trigger points and inner life of students with suicidal behaviors. Additionally, psychiatric diagnosis or pathology of the deceased students surveyed in this study was not based on an objective evaluation but on parent/student reports or teacher judgment. Therefore, interpreting the mental health problem question while considering these points is necessary. Nevertheless, the present findings could provide a better understanding of the psychosocial aspects of suicidal behavior in children and adolescents. Our results could also be used to develop more effective prevention, intervention, and treatment protocols for suicide in the medium to long term.

## 5. Conclusions

The present study attempted to identify characteristics for suicide and gender differences by examining data on Korean students. The findings suggest that the effective implementation of suicide warning signals and suicide-related mental health education for teachers could contribute to the prevention of suicide in children and adolescents. A further study should include additional data such as parental observation with a healthy control to identify factors for improving suicidality prediction, prevention, and interventions in children and adolescents.

## Figures and Tables

**Table 1:** Sociodemographic characteristics of students who died by suicide in 2016–2020 (N = 654).

Variables		N (%)
Age	M * = 16.0 (SD * = 1.7)	
Gender	Boys	344 (52.6)
	Girls	310 (47.4)
School type	Elementary school	25 (3.8)
	Middle school	204 (31.2)
	High school	425 (65.0)
Family structure	Living together	460 (73.2)
	Separation or divorce	146 (22.3)
	Dead (one or both)	22 (3.5)
Socioeconomic status	High	55 (10.2)
	Middle	359 (66.4)
	Low	127 (23.5)
Place of suicide	Home	401 (62.1)
	Public space	149 (23.1)
	Hospital	14 (2.2)
	School	14 (2.2)
	Other	68 (10.5)
Method of suicide	Jumping from a high place	454 (70.6)
	Hanging	165 (25.7)
	Drug poisoning	6 (0.9)
	Gas poisoning	12 (1.9)
	Other	6 (0.9)
Psychological problems perceived by teachers	Yes	247 (42.1)
	No	340 (57.9)
Physical illness	Yes	112 (18.7)
	No	486 (81.3)
Mental illness	Yes	138 (24.4)
	No	427 (75.6)
Trigger event before suicide	Yes	252 (47.9)
	No	274 (52.1)
History of attempted suicide	Yes	48 (9.4)
	No	465 (90.6)
History of self-harm	Yes	73 (13.4)
	No	470 (86.6)
Experience of an acquaintance’s death	Yes	45 (13.0)
	No	300 (87.0)
Experience of an acquaintance’s death by suicide	Yes	48 (12.8)
	No	328 (87.2)
Warning sign of suicide	Yes	120 (20.6)
	No	462 (79.4)
Attendance	Good	456 (54.2)
	Dropped out or absent due to illness	233 (27.7)
	Dropped out or absent without permission	141 (16.8)
	Others	11 (1.3)
School acceptance	M * = 3.05, SD * = 0.8	

* M = Mean, SD = Standard deviation.

**Table 2:** Usual concerns revealed at school within one year prior to the death of a student who died by suicide in 2016–2020 (N = 654).

Variables		N (%)
Individual problems	Appearance complex	41 (6.3)
	Physical problems	47 (7.2)
	Mental health problems	121 (18.5)
	Other	23 (3.5)
	None	422 (64.5)
Family problems	Domestic violence	14 (2.1)
	Financial difficulties	64 (9.8)
	Physical problems of family members	15 (2.3)
	Mental health problems of family members	22 (3.4)
	Conflict between parent and child	155 (23.7)
	Conflict between parents	47 (7.2)
	Others	35 (5.4)
	None	302 (46.2)
Peer problems	Victimization of extortion/violence	7 (1.1)
	Perpetration of extortion/violence	10 (1.5)
	Bullying/cyberbullying	4 (0.6)
	Joining school gang	1 (0.2)
	Problems related to dating	43 (6.6)
	Conflict between peers	13 (2.0)
	Other	39 (6.0)
	None	537 (82.1)
Academic problems	Poor grades and low academic performance	67 (10.2)
	Pessimism about dropping grades	11 (1.7)
	Burden of competitive school environment	9 (1.4)
	Burden of learning workload	19 (2.9)
	Burden of learning and future career	77 (11.8)
	Fear of academic failure	34 (5.2)
	Parental pressure concerning high grades	44 (6.7)
	Indifference to learning	15 (2.3)
	Other	29 (4.4)
	None	349 (53.4)
Addiction problems	Gaming addiction	40 (6.1)
	Internet addiction	14 (2.1)
	Smartphone addiction	36 (5.5)
	Pornography addiction	3 (0.5)
	Drug addiction	1 (0.2)
	Gambling addiction	2 (0.3)
	Other	13 (2.0)
	None	545 (83.3)

**Table 3:** Comparison of gender differences of students who died by suicide in 2016–2020 (N = 654).

Variables					
			Boys (N = 334)	Girls (N = 310)	t/X^2^-Value
Age (year)	-		16.0 (1.79)	16.0 (1.68)	0.223 (df = 648)
School type	Elementary school		17 (4.9)	8 (2.6)	2.744 (df = 2)
	Middle school		103 (29.9)	101 (32.6)	
	High school		224 (65.1)	201 (64.8)	
Parents	Living with together		251 (74.5)	209 (70.1)	2.869 (df = 2)
	Separation of divorce		74 (22.0)	79 (26.5)	
	Dead (one or both)		12 (3.6)	10 (3.4)	
Socioeconomic status	High		30 (10.7)	25 (9.6)	1.066 (df = 2)
	Middle		190 (67.6)	169 (65.0)	
	Low		61 (21.7)	66 (25.4)	
Place of suicide	Home		209 (61.3)	192 (63)	8.709 (df = 4)
	Public space		87 (25.5)	62 (20.3)	
	Hospital		3 (0.9)	11 (3.6)	
	School		9 (2.6)	5 (1.6)	
	Other		33 (9.7)	35 (11.5)	
Method of suicide	Jumping from a high place		229 (67.6)	225 (74.0)	15.607 (df = 4) ***
	Hanging		97 (28.6)	68 (22.4)	
	Drug poisoning		0 (0.0)	6 (2.0)	
	Gas poisoning		7 (2.1)	5 (1.6)	
	Other		6 (1.8)	0 (0.0)	
Psychological problems perceived by teachers	Yes		111 (35.1)	136 (50.2)	13.572 (df = 1) ***
	No		205 (64.9)	135 (49.8)	
Physical illness	Yes		55 (16.8)	57 (21.0)	1.729 (df = 1)
	No		272 (83.2)	214 (79.0)	
Mental illness	Yes		48 (15.5)	90 (35.3)	29.746 (df = 1) ***
	No		262 (84.5)	165 (64.7)	
Trigger event before suicide	Yes		150 (53.0)	102 (42.0)	6.371 (df = 1) *
	No		133 (47.0)	141 (58.0)	
History of attempted suicide	Yes		11 (3.8)	37 (16.3)	23.141 (df = 1) ***
	No		275 (96.2)	190 (83.7)	
History of self-harm	Yes		11 (3.7)	62 (25.2)	53.448 (df = 1) ***
	No		286 (96.3)	184 (74.8)	
Experience of an acquaintance’s death	Yes		26 (14.4)	19 (11.6)	0.586 (df = 1)
	No		155 (85.6)	145 (88.4)	
Experience of an acquaintance’s death by suicide	Yes		20 (10.1)	28 (15.8)	2.80 (df = 1)
	No		179 (89.9)	149 (84.2)	
Warning sign of suicide	Yes		50 (16.1)	70 (25.8)	8.416 (df = 1) **
	No		261 (83.9)	201 (74.2)	
Attendance	Good	Yes	257 (75.1)	199 (66.1)	6.334 (df = 1) *
		No	85 (24.9)	102 (33.9)	
	Dropped out or absent due to illness	Yes	62 (18.1)	97 (32.2)	17.093 (df = 1) ***
		No	280 (81.9)	204 (67.8)	
	Dropped out or absent without permission	Yes	60 (17.5)	42 (14.0)	1.546 (df = 1)
		No	282 (82.5)	259 (86.0)	
	Other	Yes	4 (1.2)	7 (2.3)	1.272 (df = 1)
		No	338 (98.8)	294 (97.7)	
School acceptance			3.07 (0.808)	3.03 (0.888)	0.522 (df = 528)

Note: * *p* < 0.05, ** *p* < 0.01, *** *p* < 0.001.

**Table 4:** Comparison of gender differences in concerns within 1 year of students’ deaths by suicide in 2016–2020 (N = 654).

Variables		Boys		Girls		X^2^-Value
		Yes	No	Yes	No	
Individual problems	Appearance complex	16 (5.4)	279 (94.6)	25 (9.2)	246 (90.8)	3.038 (df = 1)
	Physical problems	20 (6.8)	275 (93.2)	27 (10.0)	244 (90.0)	1.880
	Mental health problems	42 (14.2)	253 (85.8)	79 (29.2)	192 (70.8)	18.692 ***
	Other	17 (5.8)	278 (94.2)	6 (2.2)	265 (97.8)	4.563 *
Family problems	Domestic violence	6 (2.2)	262 (97.8)	8 (3.4)	230 (96.6)	0.590 (df = 1)
	Financial difficulties	31 (11.6)	237 (88.4)	33 (13.9)	205 (86.1)	0.603
	Physical problems of family members	5 (1.9)	263 (98.1)	10 (4.2)	228 (95.8)	2.391
	Mental health problems of family members	9 (3.4)	259 (96.6)	13 (5.5)	225 (94.5)	1.342
	Conflict between parent and child	70 (26.0)	199 (74.0)	85 (35.7)	153 (64.3)	5.588 *
	Conflict between parents	27 (10.1)	241 (89.9)	20 (8.4)	218 (91.6)	0.418
	Other	15 (5.6)	253 (94.4)	20 (8.4)	218 (91.6)	1.542
Peer problems	Victimization of extortion/violence	3 (1.0)	306 (99.0)	4 (1.5)	259 (98.5)	0.356 (df = 1)
	Perpetration of extortion/violence	7 (2.3)	302 (97.7)	3 (1.1)	260 (98.9)	1.046
	Bullying/ cyberbullying	1 (0.3)	308 (99.7)	3 (1.1)	260 (98.9)	1.366
	Joining school gang	1 (0.3)	308 (99.7)	0 (0.0)	263 (100.0)	0.853
	Problems related to dating	20 (6.5)	289 (93.5)	23 (8.7)	240 (91.3)	1.056
	Conflict between peers	4 (2.9)	136 (97.1)	9 (8.0)	104 (92.0)	3.346
	Other	18 (5.8)	291 (94.2)	21 (8.0)	242 (92.0)	1.043
Academic problems	Poor grades and poor academic performance	32 (10.2)	283 (89.8)	35 (13.0)	235 (87.0)	1.127 (df = 1)
	Pessimism about dropping grades	4 (1.3)	311 (98.7)	7 (2.6)	263 (97.4)	1.379
	Burden of high competition in school	1 (0.3)	314 (99.7)	8 (3.0)	262 (97.0)	6.717 *
	Burden of learning workload	6 (1.9)	309 (98.1)	13 (4.8)	257 (95.2)	3.918
	Burden of learning and future career	36 (11.4)	279 (88.6)	41 (15.2)	229 (84.8)	1.795
	Fear of academic failure	15 (4.8)	300 (95.2)	19 (7.0)	251 (93.0)	1.375
	Parental pressure concerning high grades	22 (7.0)	293 (93.0)	22 (8.1)	248 (91.9)	0.283
	Indifference to learning	8 (6.0)	125 (94.0)	7 (6.0)	110 (94.0)	0.00
	Other	15 (4.8)	300 (95.2)	14 (5.2)	256 (94.8)	0.055
Addiction problems	Gaming addiction	36 (12.6)	249 (87.4)	4 (1.6)	239 (98.4)	22.607 ***
	Internet addiction	9 (3.2)	278 (96.8)	5 (2.1)	238 (97.9)	0.615
	Smartphone addiction	23 (8.1)	262 (91.9)	13 (5.3)	230 (94.7)	1.528
	Pornography addiction	2 (0.7)	283 (99.3)	1 (0.4)	242 (99.6)	0.196
	Drug addiction	0 (0.0)	228 (100.0)	1 (0.5)	211 (99.5)	1.078
	Gambling addiction	2 (1.6)	122 (98.4)	0 (0.0)	102 (100.0)	1.660
	Others	9 (3.2)	276 (96.8)	4 (1.6)	239 (98.4)	1.248

Note: * *p* < 0.05, *** *p* < 0.001.

## Data Availability

Not applicable.
